# *Fasciola hepatica* Infection in Cattle: Analyzing Responses of Peripheral Blood Mononuclear Cells (PBMC) Using a Transcriptomics Approach

**DOI:** 10.3389/fimmu.2019.02081

**Published:** 2019-08-29

**Authors:** Andres Garcia-Campos, Carolina N. Correia, Amalia Naranjo-Lucena, Laura Garza-Cuartero, Gabriella Farries, John A. Browne, David E. MacHugh, Grace Mulcahy

**Affiliations:** ^1^UCD School of Veterinary Medicine, University College Dublin, Dublin, Ireland; ^2^Animal Genomics Laboratory, UCD School of Agriculture and Food Science, University College Dublin, Dublin, Ireland; ^3^UCD Conway Institute of Biomolecular and Biomedical Research, University College Dublin, Dublin, Ireland

**Keywords:** *Fasciola*, transcriptomics, vaccines, immunoregulation, cattle, PBMCs, apoptosis

## Abstract

The parasitic helminth *Fasciola hepatica* (liver fluke) causes economic loss to the livestock industry globally and also causes zoonotic disease. New control strategies such as vaccines are urgently needed, due to the rise of drug resistance in parasite populations. Vaccine development requires a comprehensive understanding of the immunological events during infection. Previous *in vivo* studies by our group have investigated global differentially expressed genes (DEGs) in ovine peripheral blood mononuclear cells (PBMC) in response to both acute and chronic *F. hepatica* infection. This work demonstrated that pathways involved in the pathogenesis of ovine fasciolosis included fibrosis, inhibition of macrophage nitric oxide production, and antibody isotype switching, among others. Transcriptomic changes in PBMC populations following *F. hepatica* infection in cattle, in which the disease phenotype is quite different, have not yet been examined. Using RNA sequencing we investigated gene expression changes in PBMC isolated from 9 non-infected and 11 *F. hepatica*-experimentally-infected calves immediately before infection, at 1 and at 14 weeks post-infection. Longitudinal time-course comparisons between groups revealed 21 and 1,624 DEGs driven exclusively by *F. hepatica* infection in cattle at acute and chronic stages, respectively. These results show that fewer DEGs at the acute stage of infection can be identified in cattle, as compared with sheep. In addition, the log_2_ fold-changes of these DEGs were relatively low (−1 to 3) reflecting the different clinical presentation of *F. hepatica* infection in cattle. Gene pathways for hepatic fibrosis and hepatic cholestasis along with apoptosis of antigen-presenting cells were enriched at chronic stages. Our results reflect the major differences in the disease phenotype between cattle and sheep and may indicate pathways to target in vaccine development.

## Introduction

The liver flukes *Fasciola hepatica* and *Fasciola gigantica* are parasitic trematodes that affect cattle, sheep and goats, worldwide causing significant economic loses to agriculture (1, 2). These parasites also infect people, with most human fasciolosis cases concentrated in South America, Africa and Asia (3), although human fasciolosis has also been reported in other areas such as Turkey (4), Serbia (5), Denmark (6), and Germany (7). In addition, to ruminants and humans a wide range of other mammalian species can be infected.

*Fasciola hepatica* infection of the definitive host occurs after ingestion of metacercariae, the infective stage, dispersed on pasture. Once the metacercariae are in the small intestine, newly excysted juveniles (NEJs) hatch, and migrate via the peritoneum for 4–6 days (early stages of infection) until they reach the liver capsule. In the liver, juvenile flukes commence migrating and feeding throughout the liver parenchyma. In this phase, haemorrhagic tracts and also the early stages of tissue repair can be seen. Eight to ten weeks later, the flukes reach the bile ducts, where they become mature and commence egg-laying. For several decades, triclabendazole has been the drug of choice for the clearance of *F. hepatica* infection as it can target early immature, immature and mature flukes, unlike other flukicides. Nevertheless, the use of this drug is restricted for dairy cattle in certain countries. In addition, an increase in triclabendazole-resistant liver fluke populations is evident (8), and presents difficulties for the control of fasciolosis in farmed ruminants. Accordingly, several research projects are pursuing alternative prophylactic and therapeutic strategies including new active compounds (9) and vaccines (10). The latter are especially desirable as vaccines could reduce the use of flukicides, thus slowing the emergence of anthelmintic resistance in the future, and do not leave drug residues that present risks both to the environment and to consumers of animal products.

In order to develop an effective vaccine strategy, it is important to understand the key immunological features that the parasite triggers in the mammalian host. It has been shown that stimulation with liver fluke-derived extracts, such as excretory/secretory (ES) products and tegument, induce unbalanced Th1/Th2 responses (11–13), alternative activation of macrophages (14) and cell death (15). In addition, glycoconjugates derived from liver fluke extracts induce activation of peritoneal eosinophils (16) and apoptosis of macrophages (17). Transcriptional analysis revealed up-regulated *TGFB1* following *F. hepatica* infection in ovine liver (18) and peripheral blood mononuclear cells (PBMC) (19, 20). This is consistent with the fibrosis seen in chronic infection in sheep, although there are indications that by 14 weeks post-infection this is limited via *SMAD7* down-regulation (20). Sheep transcriptome studies also revealed differentially expressed genes (DEGs) involved in T-cell activation (19), activation of the death receptor signaling pathway and the inhibition of nitric oxide production in macrophages along with Toll-like receptor signaling pathways after *F. hepatica* infection (20, 21).

The use of RNA sequencing (RNA-seq) has also been useful in clarifying the underlying mechanisms involved in the immunology of fasciolosis, and in particular, how immunoregulation induced by the parasite can be overcome. For example, it has been hypothesized that the IL-18 and IL-12 complex, two important up-stream regulators, are inhibited during *F. hepatica* infection of sheep, and this could be responsible for switching the humoral response toward the production of IgG1 antibodies rather than IgG2 (20), as is seen during infection (22). In line with this observation, it has been shown that the involvement of genes associated with nitric oxide production, IL-12 signaling and IL-8 signaling could confer protective immunity when mice were vaccinated with T-cell epitopes derived from *F. hepatica* cathepsin B (23).

Cattle, along with sheep, are the primary target for a liver fluke vaccine. The disease phenotype in cattle differs significantly from that in sheep. Whereas, cattle may exhibit delayed growth and loss of productivity due to chronic infection, sheep can suffer from sudden death following heavy infection (24), as well as acute disease. Accordingly, we set out to carry out transcriptomic analysis of PBMC during the acute and chronic stages of *F. hepatica* infection in cattle, and to compare this with similar studies previously performed in sheep.

## Materials and Methods

### Ethics, Experimental Design, and Group Size Calculation

Animals and procedures carried out were approved by the UCD Animal Research Committee (AREC-16-23-Mulcahy) and licensed by the Health Products Regulatory Authority (AE18982/P098). Twenty castrated male Holstein-Friesian and crossbred Holstein-Friesian calves, 3–6 months of age, obtained from a small number of local farms with no history of *F. hepatica* infection were included in the study. Animals were divided into uninfected (*n* = 9; 4 Holstein-Friesian and 5 crossbred Holstein-Friesian) and infected (*n* = 11; 6 Holstein-Friesian and 5 crossbred Holstein-Friesian) groups and housed at UCD Lyons Research Farm throughout the experimental period. Calculation of the minimum number of animals required was based on Hart et al. (25) considering the following factors: two-tailed test with 90% power, minimum depth of coverage of 10 million reads per sample, fold change in expression between groups equal to 2, and the coefficient of variance of gene expression as an average of the variance of gene expression seen in the bovine atlas (26). Inclusion of animals in one group or another was based on blocking by age and breed in order to reduce confounding.

Animals were housed in two adjacent pens from 3 weeks prior to infection (W-3) to 15 weeks (W15) post-infection. In order to reduce pen housing as a confounding factor, animals from both uninfected and infected groups were mixed and allocated to each pen, while each also contained 5 Holstein-Friesian and 5 crossbred Holstein-Friesian animals. Throughout the experimental period animals were fed silage and had access to water *ad libitum*. Health checks were conducted daily during the study.

### Experimental Infection

Sedimentation performed on fecal samples for *F. hepatica* eggs, and ELISA testing for the absence of *F. hepatica* cathepsin L1 (CL1) antibodies in sera as previously described (22) were used to demonstrate that animals were free from *F. hepatica* infection at the beginning of the study (W-3). Three weeks later (W0), each animal in the infected group was orally challenged with 200 *F. hepatica* metacercariae, Italian strain (Ridgeway Research Ltd, UK) suspended in 10 mL of distilled water. Animals in the uninfected group received 10 mL distilled water only. The viability of metacercariae was verified by excystation tests as described elsewhere (27).

### Sampling Procedures

Blood samples were collected by jugular venepuncture at the day of infection (W0), but before the animals were infected; then at 1 week (W1) and at 14 weeks (W14) post-infection; using plain, EDTA, and lithium heparin-coated vacutainers. Clotted samples were centrifuged at 2,300 × *g* for 5 min, and the serum subsequently removed and frozen at −20°C until ELISA testing. Antibodies specific for *F. hepatica* CL1 were detected by ELISA as previously described (22). EDTA samples were used to obtain total and differential white cell counts using an ADVIA® 2120 hematology analyser (Siemens). Fecal samples were collected at W14 in order to detect fluke eggs, nematode eggs and lungworm larvae, using Sedimentation, McMaster and modified Baermann testing, respectively.

### PBMC Isolation and RNA Extraction

Blood collected in lithium heparin tubes was used for PBMC isolation as previously described (20) with the substitution of 15 mL of Ficoll-Paque PLUS (GE Healthcare) for Histopaque 1.077 g/mL. Following evaluation of cell viability by trypan blue staining and cell counts, aliquots of 2 × 10^7^ PBMC per sample were washed and suspended in 600 μL of RLT buffer (Qiagen) supplemented with 10 μL/mL of β-mercaptoethanol. Cells were disrupted immediately by vortexing for 30 s and stored at −80°C. Total RNA was isolated using the RNeasy Plus Mini Kit (Qiagen) following the manufacturer's instructions. This kit includes a genomic DNA eliminator column. The quantity of RNA extracted was measured using a Nanodrop 1000 spectrophotometer (Thermo Fisher Scientific), and RNA quality was evaluated using an Agilent 2100 Bioanalyzer with an RNA 6000 Nano Lab Chip Kit (Agilent Technologies). All samples exhibited A260/280 ratio >2.0 and RNA integrity number (RIN) >8.

### Sacrifice of Animals and Post-mortem Examination

At week 15, animals were sent to the abattoir, and livers were recovered. After gross examination of the liver parenchyma and the bile ducts the liver fluke burden was estimated for each animal as previously described (28). Mean fluke numbers collected from Holstein-Friesian and crossbred Holstein-Friesian calves of the infected group were compared with the Wilcoxon rank-sum test.

### RNA Preparation and Sequencing

RNA-seq library preparation and sequencing was performed by the Brigham Young University (BYU) DNA Sequencing Center (https://dnasc.byu.edu, UT, USA). Sixty barcoded paired-end (PE) mRNA-seq libraries were prepared using a KAPA stranded mRNA-seq kit and KAPA Dual-Indexed Adapter Kit (Roche), following the manufacturer's instructions. After quality control, a pool of all 60 barcoded samples was loaded across 8 lanes of v4 chemistry high output flow cells at 2 × 125 nucleotide reads on an Illumina HiSeq 2500 Sequencing System. All RNA-seq data has been made available *via* the European Nucleotide Archive repository under the accession number PRJEB32022.

### Differential Expression Analysis

Bioinformatics analyses were performed using scripts developed in GNU bash version 4.3.48 (http://ftp.gnu.org/gnu/bash), and R version 3.5.1 (29). All scripts can be accessed at a public GitHub repository (https://github.com/carolcorreia/Liver-Fluke-RNAseq). Deconvoluted FASTQ files were evaluated with FastQC v0.11.5 (www.bioinformatics.babraham.ac.uk/projects/fastqc). After filtering out adapter sequences, removing poor quality reads (those where more than 25% of the nucleotides had a Phred Score (Q) lower than 20) and removing PE reads which lengths were shorter than 100 nucleotides with ngsShoRT v2.2 (30), the clean FASTQ files were re-assessed with FASTQC as before. Filtered PE reads were then aligned and annotated to the bovine genome (Bos taurus_ ARS-UCD1.2) using the alignment software STAR v2.6.0c (31). After the 2-pass alignment and elimination of unmapped and multimapping reads, the gene counts were generated using the package featureCounts from the Subreads v1.5.1 software package (32). In addition, for each sample, filtered reads were subjected to variant calling for the evaluation of SNPs. The identity of samples was re-confirmed by the identity-by-state (IBS) comparison with PLINK v1.9 (33). The Z2 values between two samples were ranked and the first 60 Z2 values were used to verify whether they belonged to the same animal.

**Figure 1:**
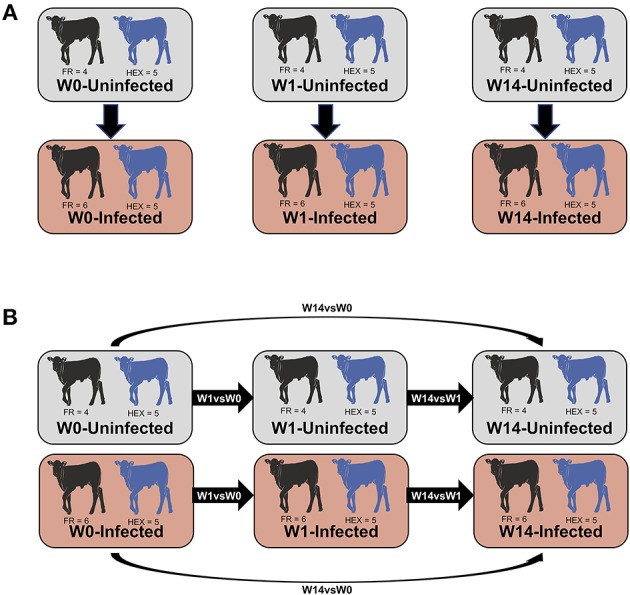
Summary of the comparisons used for the differential expression analysis. Non-paired comparisons between uninfected and infected groups at week 0 (W0), 1 week (W1), and 14 weeks (W14) post-experimental infection **(A)**. Longitudinal time-course analysis of DEGs for each group between week 1 and week 0 (W1 vs. W0), week 14 and week 1 (W14 vs. W1), week 14 and week 0 (W14 vs. W0) **(B)**. DEGs obtained exclusively in the infected group were employed for IPA downstream analysis.

Prior to normalization of the libraries, lowly-expressed genes with <1 count per million in 9 or more libraries were discarded from further analysis. Normalization factors were then calculated using the trimmed mean of M-values with the R/Bioconductor EdgeR package v3.22.3 (34, 35). The overall data structure was reviewed with multidimensional scaling (MDS) at each time point to detect potential outliers. Two approaches were used for identification of DEGs (Figure 1A) non-paired comparisons between uninfected and infected groups at each time point and (Figure 1B) longitudinal time-course analysis of DEGs for each group and then selecting those obtained exclusively in the infected group. Model matrices were generated for each comparison. For the first approach, infection status and time point were modeled, including interaction and blocking by breed. For the second approach, separate design matrices were generated for infected vs. non-infected animals, examining interactions between infection status and time, whilst blocking by animal. For both approaches, common and trended dispersions were estimated using the Cox-Reid profile-adjusted likelihood function (36) and tagwise dispersion was estimated with the empirical Bayes method to fit negative binomial generalized linear models. DEGs were determined using the quasi-likelihood *F*-test (37) choosing a false discovery rate (FDR) cut-off of 5%.

### Ingenuity Pathway Analysis of Differentially Expressed Genes

Genes identified as differentially expressed exclusively in the infected group using the second time course approach (Figure 1B) were analyzed using Ingenuity Pathway Analysis (IPA; Qiagen Inc., www.qiagenbioinformatics.com/products/ingenuitypathway-analysis). Human orthologs of bovine genes were searched and merged using the HGNC Comparison of Orthology Predictions (HCOP) (38). Where a bovine gene was annotated to multiple human genes just one human ortholog was used for the analysis. Detection of significant functional canonical pathways was established when the enrichment *P*-value was <0.05.

The IPA “upstream regulators” and “causal network” features were used to analyse upstream and master regulators. The Ingenuity Knowledge Base was used to predict the expected causal effects between upstream regulators and DEG targets. Ingenuity Knowledge Base was also used to predict the expected downstream effects due to the DEG targets by using the “disease and function” and “regulatory effect” functions. Hits were considered significant when the *P*-value of overlap was lower than 0.05. Z-score values > +2 or below −2 were used to predict the activation or inhibition status of the upstream and master regulator or of the increased or decreased representation of a disease or functions, as appropriate.

### PCR Validation

Six genes involved in immunological or signaling pathways (*IL17RB, IL1B, TNF, TGFB2, PRG3*, and *NOS2*) were selected for validation using reverse transcription quantitative real-time PCR (RT-qPCR). For all 60 samples, cDNA was synthesized with 1.0 μg of total RNA from each sample used for the RNA-seq library preparation. The High-Capacity cDNA Reverse Transcription Kit (Applied Biosystems) was used for this purpose according to the manufacturer's instructions. After cDNA synthesis the 20.0 μL reaction was diluted 1:20 with distilled H_2_O prior to use. Primers were designed based on the corresponding cDNA sequences (obtained from the Ensembl database), and primer efficiencies were determined using a 1:4 dilution series over 7 points. PCR amplification specificity was assessed using melting curve analysis to confirm the presence of a single sharp peak. RT-qPCR was performed using SYBR Green Master Mix (Applied Biosystems) on a 7500 Real-Time PCR System (Applied Biosystems). All samples were analyzed in duplicate. Each 20 μL PCR reaction contained 5.0 μL diluted cDNA samples (or appropriate controls), 10.0 μL of SYBR Master Mix and 300 nM final concentration of each primer pair. Each RT-qPCR reaction was performed using the following cycling parameters: 2 min at 95°C (heat-activation step); 40 cycles of 15 s at 95°C, 1 min at 60°C. A dissociation curve was also included. The stability of eight potential reference genes (*ACTB, GAPDH, H3F3A, PPIA, RPL19, RNF11, SDHA*, and *YWHAZ*) was assessed using the GeNorm algorithm within the qBase+ software package (Biogazelle) (39, 40). *H3F3A* and *RPL19* were shown to be the most stably expressed reference genes (M <0.14) and were used for subsequent normalization. Calibrated normalized relative quantities were calculated using the qBase+ software. Two-tailed paired sample *t*-tests were used to identify statistically significant gene expression changes using the same software.

## Results

### Confirmation of *F. hepatica* Infection by ELISA, Sedimentation, and Fluke Burden

**Figure 2:**
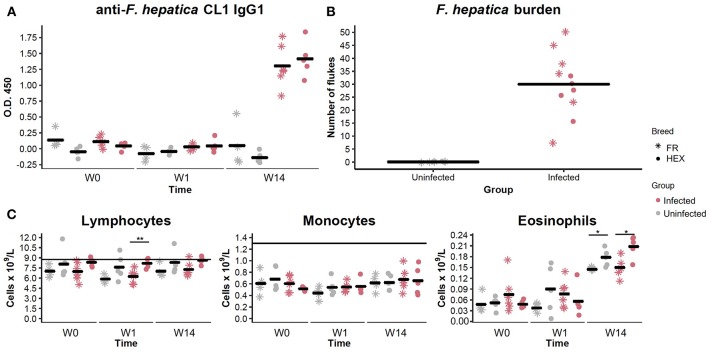
Evaluation of anti *Fasciola hepatica* Cathepsin L1 IgG1 antibodies in uninfected (shapes in gray) and infected (shapes in pink) animals by ELISA at week 0 (W0), 1 week (W1), and 14 weeks (W14) post-experimental infection. Each dot and star represents optical density (O.D.) values at 450 nm for each Holstein-Friesian crossbred and Holstein-Friesian individual, respectively. Horizontal lines represent the mean for each group and time point **(A)**. Fluke burden found in livers and bile ducts of uninfected and infected animals 15 weeks post-infection. Each star represents numbers of *F. hepatica* flukes for each Holstein-Friesian animal. Each dot represents numbers of *F. hepatica* flukes for each Holstein-Friesian crossbred animal. Horizontal lines represent the mean for each group **(B)**. Dynamics of the different PBMC sub-populations (lymphocytes, monocytes, and eosinophils) in uninfected (gray) and infected (pink) animals at W0, W1, and W14. Each dot and star represent absolute values that are expressed in cells × 10^9^/L for each Holstein-Friesian crossbred and Holstein-Friesian individual, respectively. Horizontal line represents the mean for each group and time point. Dots and stars located above the horizontal line indicate that values are above the standard reference range **(C)**. ^*^P = 0.08 ^**^P = 0.009.

Prior to the beginning of the experimental infection, all the animals were healthy and showed no signs of disease. Baermann tests were negative for all animals at W0 and W14 ruling out the presence of lungworm infection during the experimental period. Two animals, one from each group, were observed to have nematode eggs in their feces, with fecal egg counts (FECs) of 50 strongyle eggs per gram at W0. None of the animals showed evidence of anti-*F. hepatica* CL1 IgG1 antibodies or presence of liver fluke eggs at W0. After challenge with *F. hepatica* metacercariae, with viability and excystation confirmed by a 65% successful hatching rate *in vitro, F. hepatica* eggs were detected in nine out of eleven animals belonging to the infected group at W14. All 11 animals in the infected group seroconverted, as determined by ELISA, with O.D. values above the cut-off at W14 (Figure 2A).

When livers were recovered, all the infected animals were shown to harbor adult and immature *F. hepatica* specimens in bile ducts and liver parenchyma. Individual liver fluke counts are shown in Figure 2B. No significant differences were detected in fluke numbers between Holstein-Friesian and crossbred Holstein-Friesian animals of the infected group (Mann-Whitney U, *P* = 0.405). Hematological parameters were within the reference range for all the animals at W0. There were no significant differences in the numbers of lymphocytes, monocytes, and eosinophils between uninfected and infected groups at any time point. There was an increase in the eosinophil counts between W1 and W14 in both groups (Figure 2C), but values remained within the reference range. Lymphocyte numbers in infected crossbred Holstein-Friesian at W1, and eosinophil numbers in both infected and control crossbred Holstein-Friesian animals at W14, were significantly greater than comparator cohorts (Figure 2C). Infected animals showed thickening of bile ducts, fibrotic areas, and haemorrhagic tracts in the liver (Supplementary Figure S1).

### Bioinformatics and Mapping to the Bovine Genome

Sequencing of the pooled RNA-seq libraries across the 8 lanes generated a mean of 29.94 million raw reads per library. From these, 87.72% (~26.24 million reads) of the reads passed the filtering steps, and a mean of 21.82 million reads uniquely mapped to the bovine genome (Supplementary Table S1). Calculation of the IBS distance between samples confirmed the correct labeling of samples throughout the experiment. Z2 values calculated between samples from different individuals were lower than 0.43 (Supplementary Table S2). Post-alignment quality control showed that libraries from all samples displayed a similar density of gene counts (Supplementary Figure S2).

**Figure 3:**
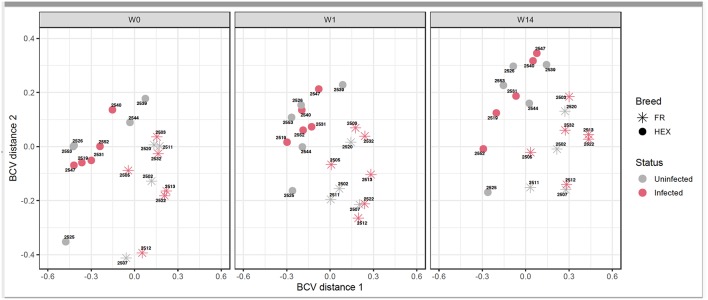
Multidimensional scaling of uninfected and infected Holstein-Friesian and Holstein-Friesian crossbred animals at week 0 (W0), week 1 (W1), and week 14 (W14) post *F. hepatica* infection. The BCV distance 1 (x-axis) and 2 (y-axis) represent the directions that separate the gene expression of the samples to the greatest extent.

Following the two approaches used for identification of DEGs (Figure 1A) non-paired comparisons between uninfected and infected groups at each time point and (Figure 1B) longitudinal time-course analysis of DEGs for each group and then selecting those obtained exclusively in the infected group, different insights were obtained. MDS showed no separation between uninfected and infected animals after *F. hepatica* infection at any time point. As shown in the MDS plot (Figure 3), there were clear overlaps between animals from uninfected and infected groups at each of the time-points, suggesting that infection was not the largest source of variation among animals. When the MDS plot was re-analyzed including breed as an additional factor, there was a clear separation between Holstein-Friesian and Holstein-Friesian crossbred cattle on the x-axis (BCV distance 1). Indeed, there were 3,410 DEG identified between Holstein-Friesians and crossbred Holstein-Friesian at W0 (Supplementary Table S3). Because breed was considered in the experimental design, sub-groups of each breed were analyzed separately in the MDS plot. Again, there was no separation between groups at W1 and W14, suggesting again that *F. hepatica* infection might not have had the largest impact on gene expression within PBMC.

### Longitudinal Time-Course Contrasts Conducted Between Time-Points in Uninfected and Infected Groups Showed the Largest Numbers of DEGs at W14

**Figure 4:**
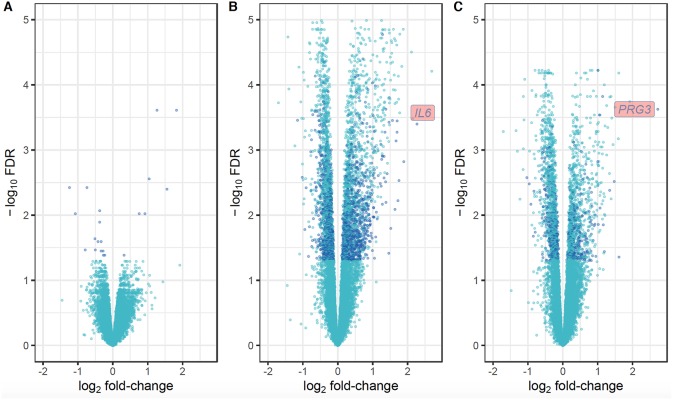
Volcano plots showing the extent of log_2_ fold-change (x-axis) and their −log_10_ FDR values (y-axis) for the DEGs identified in PBMC and their direction of expression driven specifically by *F. hepatica* infection (dark blue). Genes that did not pass the FDR threshold and those DEGs that were also identified in the uninfected group are represented in light blue. Comparison of DEGs at W1 vs. W0 **(A)**, W14 vs. W0 **(B)**, and W14 vs. W1 **(C)**.

For the first approach (Figure 1A), there was no distinction of DEGs between uninfected and infected animals at any time-point. Using the second approach (Figure 1B), which overcomes individual variability, we identified 21, 3,992 and 2,324 DEGs at the W1 vs. W0, W14 vs. W0, and W14 vs. W1 comparisons, respectively. The list of these DEGs is provided in Supplementary Table S4. After performing the same comparison in the uninfected group and removing the DEGs that overlapped in both groups for each of the time points (Supplementary Figure S3), 21, 1,624, and 551 DEGs were found exclusively in the infected group at W1 vs. W0, W14 vs. W0, and W14 vs. W1, suggesting that these are DEGs driven exclusively by *F. hepatica* infection (Supplementary Table S5). RT-qPCR results for the six genes selected confirmed the significance and trend of expression (Supplementary Table S6). For all comparisons, the magnitude of change in gene expression was relatively low. The log_2_ fold change (log_2_FC) values for the DEGs varied from −1.15 (FDR = 3.5E-04), for the nebulin gene (*NEB*) at the W14 vs. W0 comparison to +3.2 (FDR = 7.3E-05) for the proteoglycan 3-like gene (*LOC781990*) at the W14 vs. W1 comparison (Figure 4).

**Figure 5:**
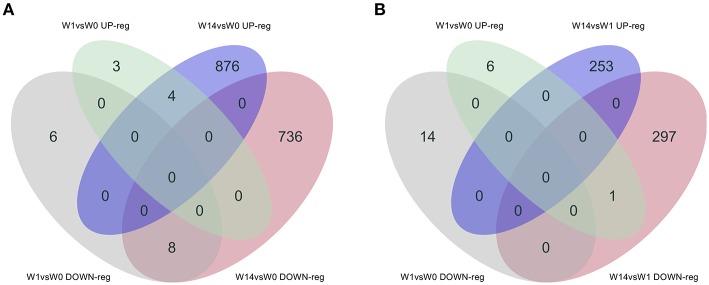
Venn diagrams showing the numbers of DEGs identified in PBMC and their direction of expression driven specifically by *F. hepatica* infection. Comparison of DEGs from W1 vs. W0 with W14 vs. W0 **(A)** and W1 vs. W0 with W14 vs. W1 **(B)**. Downregulated genes are shown in gray and pink, upregulated genes in green and blue.

As shown in the Venn diagram (Figure 5A) there were 4 genes upregulated and 8 genes downregulated common to animals during both the acute (W1) and chronic phases (W14) of the infection compared to W0. These included the T-cell receptor alpha chain V region CTL-L17-like (*LOC104970757*), interleukin 1 receptor-like 1 (*IL1RL1*), choline dehydrogenase (*CHDH*), and the antioxidant 1 copper chaperone (*ATOX1*) genes, which were upregulated, and the transportin 2 (*TNPO2*), TGFB induced factor homeobox 1 (*TGIF1*), Ras association domain family member 1 (*RASSF1*), tubulin beta 2A class IIa (*TUBB2A*), LIM domains containing 1 (*LIMD1*), HIC ZBTB transcriptional repressor 1 (*HIC1*), TNF receptor superfamily member 13C (*TNFRSF13C*) and the SHC binding and spindle associated 1 like (*SHCBP1L*) genes, which were downregulated. As summarized in Table 1, although changes in the expression of these genes were already detected at the acute stage their expression did not change significantly during the course of the infection except for the T-cell receptor alpha chain V region CTL-L17-like gene. Indeed, this was the only gene whose expression significantly decreased from the acute to the chronic phase of infection (W1: log_2_FC = 1.26, FDR = 2.5E-04; W14: log_2_FC = 0.62, FDR = 6.6E-03; Figure 5B). Other DEGs such as the proteoglycan 3 gene (*PRG3*), the previously mentioned proteoglycan 3-like, arachidonate 15-lipoxygenate gene (*ALOX15*), the C-C motif chemokine ligand 20 gene (CCL20), interleukin genes (*IL1B, IL2RA*, and *IL4I1*) and nitric oxide synthase gene (*NOS2*) were also upregulated at this stage. The DEGs identified at the W14 vs. W0 comparison included upregulated interleukin genes (*IL6, IL1B*), *TLR4*, C-C chemokine ligand genes (*CCL20, CCL3, CCL2, CCL8, CCL4*), colony stimulation factor genes (*CSF2*) the transforming growth factor beta induced gene (*TGFBI*), tumor necrosis factor gene (*TNF*), *COL7A1, COL14A1*, and *PLOD1*, among others.

**Table 1:** Common DEGs identified in PBMC at acute (W1 vs. W0) and chronic (W14 vs. W0) stages of *Fasciola hepatica* infection in cattle.

**gene_symbol**	**gene_name**	**logFC W1 vs. W0**	**FDR**	**logFC W14 vs. W0**	**FDR**
*LOC104970757*	T-cell receptor alpha chain V region CTL-L17-like	1.267	2.45E-04	0.613	0.007
*CHDH*	Choline dehydrogenase	0.757	0.009	0.526	0.005
*ATOX1*	Antioxidant 1 copper chaperone	0.324	0.041	0.224	0.014
*TNPO2*	Transportin 2	−0.230	0.041	−0.201	0.003
*TGIF1*	TGFB induced factor homeobox 1	−0.334	0.036	−0.323	0.001
*RASSF1*	Ras association domain family member 1	−0.376	0.009	−0.336	4.40E-04
*TUBB2A*	Tubulin beta 2A class IIa	−0.376	0.013	−0.209	0.022
*LIMD1*	LIM domains containing 1	−0.424	0.025	−0.518	8.23E-05
*HIC1*	HIC ZBTB transcriptional repressor 1	−0.501	0.034	−0.538	3.55E-04
*TNFRSF13C*	TNF receptor superfamily member 13C	−0.512	0.023	−0.471	9.28E-04
*SHCBP1L*	SHC binding and spindle associated 1 like	−0.790	0.034	−0.575	0.007

### Hepatic Fibrosis and Hepatic Cholestasis Are Some of the Canonical Pathways Detected at the Chronic Stage

The IPA analysis identified 27 and 98 enriched canonical pathways at W1 vs. W0 and W14 vs. W1, respectively, with 10 pathways in common for both comparisons (Supplementary Table S7). A list of the most relevant canonical pathways for each time-point are shown in Table 2. The 14-3-3-mediated signaling pathway, IL-10 signaling, Toll-like receptor signaling, TGF-β signaling, STAT3 pathways, IL-6 signaling and Th2 pathway were seen to be enriched for the W1 vs. W0 comparison, although their activation or inhibitory status could not be predicted. There were 8 pathways activated and 25 inhibited at W14 compared to W0. Ten canonical pathways that were also detected as enriched at W1, including IL-6, IL-10, and Toll-like receptor signaling, were also enriched at this stage. However, new enriched canonical pathways such as hepatic cholestasis and hepatic fibrosis/hepatic stellate cell activation were also identified. Within the inhibited canonical pathways were CD40, apoptosis signaling, TNFR1 signaling, hepatic growth factor (HGF) signaling and death receptor signaling. The production of nitric oxide and reactive oxygen species in macrophages had a trend toward inhibition with a z-score value very close to the threshold (−0.83).

**Table 2:** Common canonical pathways in PBMC at acute (W1 vs. W0) and chronic (W14 vs. W1, W14 vs. W0) stages of *F. hepatica* infection in cattle.

**Ingenuity canonical pathways**	**W1 vs. W0**			**W14 vs. W1**			**W14 vs. W0**		
	**–log (*p*-value)^a^**	**Ratio^b^**	***z*-score^c^**	**–log (p-value)^a^**	**Ratio^b^**	**z-score^c^**	**–log (*p*-value)^a^**	**Ratio^b^**	***z*-score^c^**
Sertoli cell-sertoli cell junction signaling	3.91	0.030	NA	4.59	0.143	NA	–	–	–
IL-6 signaling	1.72	0.018	0	3.61	0.139	−1.291	1.36	0.204	2.4
Toll-like receptor signaling	2.14	0.031	0	2.93	0.154	−0.333	–	–	–
Germ cell-sertoli cell junction signaling	3.8	0.028	NA	2.38	0.106	NA	–	–	–
PPAR signaling	1.99	0.026	0	1.85	0.115	0.333	–	–	–
IL-10 signaling	2.23	0.034	NA	1.64	0.121	NA	–	–	–
Osteoarthritis pathway	1.64	0.017	0	1.49	0.094	2.111	2.76	0.244	2.2
Breast cancer regulation by Stahmin 1	3.53	0.024	NA	1.45	0.084	NA	–	–	–
B cell activating factor signaling	2.55	0.05	0	1.35	0.125	−0.447	–	–	–
Axonal guidance signaling	2.72	0.015	NA	1.3	0.073	NA	–	–	–
14-3-3-mediated signaling	5.73	0.046	0	–	–	–	–	–	–
Phagosome maturation	4.18	0.035	NA	–	–	–	–	–	–
TGF-β signaling	2.04	0.027	0	–	–	–	–	–	–
STAT3 pathway	1.74	0.019	0	–	–	–	2.02	0.229	1.387
Th2 pathway	1.68	0.018	0	–	–	–	–	–	–
Antigen presentation pathway	–	–	–	3.72	0.235	NA	1.39	0.265	NA
CD40 signaling	–	–	–	3.19	0.155	−2.111	–	–	–
Apoptosis signaling	–	–	–	2.92	0.136	−1.265	–	–	–
Hepatic cholestasis	–	–	–	2.92	0.13	NA	2.29	0.24	NA
TNFR1 signaling	–	–	–	2.79	0.174	−2.121	–	–	–
TNFR2 signaling	–	–	–	1.9	0.172	0.447	–	–	–
Death receptor signaling	–	–	–	1.63	0.106	−1.667	–	–	–
Dendritic cell maturation	–	–	–	1.56	0.092	1.732	1.62	0.206	2.353
Production of nitric oxide and reactive oxygen species in macrophages	–	–	–	1.56	0.089	−0.832	–	–	–
HGF Signaling	–	–	–	1.47	0.095	−3.162	–	–	–
Hepatic fibrosis/hepatic stellate cell activation	–	–	–	1.41	0.097	NA	2.07	0.237	NA
Complement system	–	–	–	–	–	–	1.87	0.333	1.633
IL-4 signaling	–	–	–	–	–	–	1.35	0.215	NA

a *The probability of association of genes from our dataset with the canonical pathway by random chance alone using Fisher's exact test*.

b *The number of genes in our dataset that meets the cut-off criteria (FDR <0.05 and detected only in the infected group), divided by the total number of genes involved in that pathway*.

c *Overall activity status of the pathway. Z < -2 indicates a prediction of an overall decrease in activity while Z > 2 predicts an overall increase in the activity. NA indicates pathways that are currently ineligible for a prediction*.

### Activation of Apoptosis of Antigen-Presenting Cells Is One the Main Regulator Effects Identified in the Chronicity of *F. hepatica* Infection

**Figure 6:**
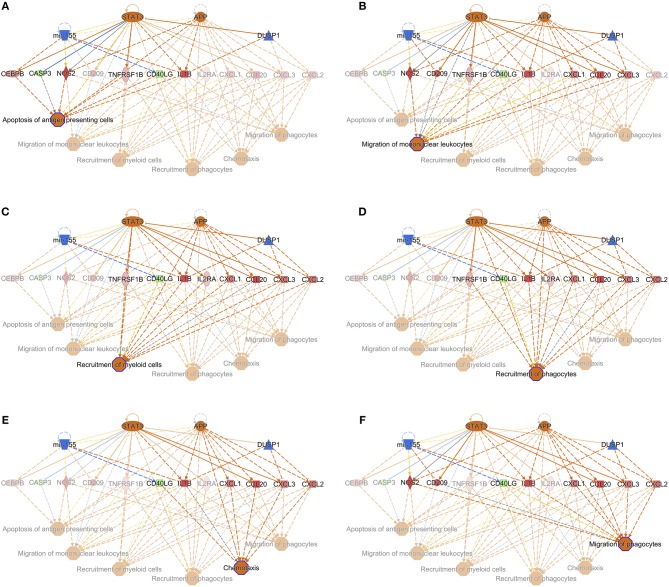
Interaction of the upstream regulators for the activation of apoptosis of antigen presenting cells **(A)**, migration of mononuclear leukocytes **(B)**, recruitment of myeloid cells **(C)**, recruitment of phagocytes **(D)**, chemotaxis **(E)**, and migration of phagocytes **(F)** in PBMC of *F. hepatica*-infected animals at the W14 vs. W1 timepoint. *STAT3* and *APP* are indicated as activated (orange), whereas mir-155 and *DUSP1* are inhibited (blue). Downstream target genes are highlighted as upregulated (red) or downregulated (green) with color intensity increasing with degree of log_2_ fold-change. Arrowheads at the end of interactions (dotted lines) indicate activation, while bars indicate inhibitory effects. The color of lines represents predicted relationships based on gene expression, including orange (activation), blue (inhibition), yellow (findings inconsistent with state of downstream molecule), and gray (effect not predicted). © 2000–2019 QIAGEN. All rights reserved.

When the presence and nature of upstream and master regulators was evaluated, that is, genes that can interfere with the transcription of other genes at different levels, IL-10 was the only upstream regulator whose z-score value (1.715) was predicted in the W1 vs. W0 comparison. It was possible to identify the activation status of 105 and 27 genes at W14 vs. W0 and W14 vs. W1 (Supplementary Table S8). The upstream regulators common to both comparisons were the activation status of IL-1B, APP, TNF, and TLR7 and the inhibition of dual specificity phosphatase 1 (DUSP1) (Table 3). IL-10 was also predicted in both comparisons with an inhibitory trend but did not reach the z-score cut-off. In terms of prediction of regulatory effects, DEGs of the W14 vs. W1 comparison clustered within 5 primary groups (Table 4). The cluster with the highest consistency score predicted that the inhibition of the master regulator DUSP1, the inhibition of the upstream regulator mir-155 and the activation of the upstream regulators STAT3 and APP lead to the downstream effects of activation of apoptosis of antigen-presenting cells, migration of phagocytes and mononuclear leukocytes, recruitment of myeloid cells and phagocytes and chemotaxis (Figure 6).

**Table 3:** Selection of upstream regulators in PBMC at chronic stages of *F. hepatica* infection.

	**W14 vs. W1**					**W14 vs. W0**				
**Upstream regulator**	**Expr log ratio**	**Predicted activation state**	**Activation z–score**	***p*–value of overlap**	**Number target genes**	**Expr log ratio**	**Predicted activation state**	**Activation *z*–score**	***p*–value of overlap**	**Number target genes**
PTGER2	0.2	Inhibited	−2.998	4.30E-11	20	–	–	–	–	–
CD40LG	−0.373		0.82	5.69E-06	31	−0.137	Activated	2.276	2.20E-06	65
Irgm1		Activated	3.148	6.02E-06	10	–	–	–	–	–
DYRK1A	−0.018	Activated	2.233	5.76E-05	5	–	–	–	–	–
NLRP3	0.621	Activated	3.079	1.09E-04	10	–	–	–	–	–
NOS2	1.611		−0.492	1.67E-04	5	0.158		−1.937	4.27E-04	7
TP53	−0.071		−1.02	2.91E-04	11	–	–	–	–	–
mir−155		Inhibited	−2.18	2.96E-03	6	–	–	–	–	–
STAT3	0.077	Activated	2.897	4.47E-03	13	−0.1		1.663	4.82E-06	35
TLR2	0.278		1.537	4.74E-03	9	0.593	Activated	4.002	5.18E-05	22
IRF1	0.076		0.254	5.68E-03	6	–	–	–	–	–
TICAM1	0.278		0.403	6.13E-03	10	−0.393	Activated	4.032	7.56E-06	27
TREM1	0.119		0.989	6.49E-03	14	0.56	Activated	2.745	3.12E-03	31
TNF	0.456	Activated	2.085	6.70E-03	22	1.415	Activated	5.799	1.35E-04	57
IL1B	0.949	Activated	2.775	7.18E-03	16	1.67	Activated	4.436	2.64E-05	43
DUSP1	0.853	Inhibited	−2.129	7.83E-03	5	0.204	Inhibited	−3.474	1.30E-05	13
APP	0.309	Activated	2.453	1.00E-02	14	0.308	Activated	3.39	1.70E-05	39
CSF1	−0.243		1.127	1.22E-02	13	–	–	–	–	–
IL6	1.058		1.658	1.46E-02	10	–	–	–	–	–
HDAC1	0.086	Activated	2.236	1.88E-02	5	–	–	–	–	–
FOXP3	0.183		−0.686	2.41E-02	6	–	–	–	–	–
CD2	−0.043	Inhibited	−2	2.42E-02	4	–	–	–	–	–
TGFB1	0.301		0.553	2.63E-02	17	0.236		−1.577	1.94E-02	40
IL10	0.254		−0.812	3.30E-02	15	0.189		−1.312	7.80E-09	55
TLR7	−0.434	Activated	2	3.41E-02	7	−0.192	Activated	3.124	7.62E-05	21
MYD88	0.021		1.943	3.94E-02	10	0.118	Activated	4.225	7.40E-08	37
IRF7	−0.233		0.103	4.85E-02	9	–	–	–	–	–
IL33		Activated	2.549	4.88E-02	12		Activated	3.807	8.27E-06	40
TLR4	–	–	–	–	–	0.756	Activated	3.16	1.88E-03	35
TLR3	–	–	–	–	–	−0.007	Activated	2.58	6.92E-03	20
TLR1	–	–	–	–	–	0.151	Activated	2.425	9.36E-04	6
TLR6	–	–	–	–	–	0.151	Activated	2.177	7.25E-03	5

**Table 4:** Prediction of regulatory effects in PMBC during chronic (W14 vs. W1) *F. hepatica* infection.

**ID**	**Consistency score**	**Node total**	**Regulators**	**Target molecules in dataset**	**Diseases & Functions**
1	12.124	22	APP, DUSP1, mir-155, STAT3	CASP3, CCL20, CD209, CD40LG, CEBPB, CXCL1, CXCL2, CXCL3, IL1B, IL2RA, NOS2, TNFRSF1B	Apoptosis of antigen presenting cells
					Chemotaxis
					Migration of mononuclear leukocytes
					Migration of phagocytes
					Recruitment of myeloid cells
					Recruitment of phagocytes
2	7.937	13	APP, DUSP1	CCL20, CXCL1, CXCL2, CXCL3, IL1B, NOS2, SOCS1	Inflammatory response
					Migration of phagocytes
					Recruitment of myeloid cells
					Recruitment of phagocytes
3	5.307	10	IL1B,TNF	CCL20, CXCL1, CXCL3, NOS2, VEGFA, VEGFC	Attraction of cells
					Migration of macrophages
4	0.5	6	PTGER2	BUB1B, KIF20A, PRC1, RACGAP1	M phase
5	−7.506	5	TLR7	CXCL2,CXCL3,IL1B	Migration of phagocytes

## Discussion

Due to the economic importance of *F. hepatica* infection in animal agriculture, and anthelmintic resistance concerns, there is an urgent need for the development of novel control methods such as vaccination. To date, inconsistent results have been obtained in ruminant vaccine trials with a wide repertoire of recombinant proteins (10). Even though considerable work has been undertaken to understand the immune response to *F. hepatica* infection, along with the immunoregulatory effects induced in infected animals, there are still many gaps in our knowledge, which could be important for the design of new vaccine strategies. In this regard, transcriptomics using technologies such as RNA-seq, represents a key tool in bridging these knowledge gaps.

To the best of the authors' knowledge this is the first transcriptomics analysis conducted in cattle (*Bos taurus*) investigating the immunological changes in PBMC following *F. hepatica* infection. The immunological effects triggered by the parasite can be detected in PBMC as “echoes” of the events occurring locally in the peritoneal cavity and the liver at the acute and chronic phases of the infection, respectively.

In this study, the phenotypic hallmarks of hepatic fibrosis and cholestasis in the chronic phases of *F. hepatica* infection in cattle were reflected in the altered gene expression changes seen at W14 in the infected animals. Although no differences in the numbers of eosinophils in peripheral blood were seen between infected and uninfected groups, *PRG3*, whose transcription and translation leads to eosinophil major basic protein, was the second most highly upregulated gene observed at the W14 compared to W1. Up-regulation of *PRG3* was also detected in ovine PBMC at 2 and 8 weeks post *F. hepatica*-infection (19) indicating a greater degree of eosinophil activation in infected animals.

It was somewhat surprising that the MDS plots in this study did not show a clear separation according to *F. hepatica* infection status. While sample identity verification tools have been widely used in humans and mice (41), no such tools have been developed for cattle as yet. Therefore, to validate that the ambiguous MDS plot results were not a consequence of sample mislabelling in our study SNPs derived from RNA-seq were used to calculate IBS distances and verify animal identity across time points. Animals were not exposed to *F. hepatica* previously and they only received one infective dose of metacercariae. It is known that small repeat infections, similar to those which occur naturally (12), produce more severe pathology than a single infective dose with equal numbers of metacercariae in cattle (42); therefore, stronger infection effect might have been observed on the MDS analysis if trickle infections had been used. MDS plots also suggest that there could be intrinsic confounders, such as breed (43), temporal changes affecting animal development, or additional environmental factors that could have had a higher impact on gene expression than infection status. This emphasizes the importance of including an uninfected time course control in these types of studies. Almost 50% of the DEGs identified at the time-course comparisons W14 vs. W0 and W14 vs. W1 were observed in both infected and uninfected animals, all of them with the same direction of expression. We consider that the use of animals aged 3–6 months of age, as a primary target group of any novel anti-fluke vaccine, was appropriate. However, it is possible that greater temporal variation in expression levels of genes related to growth and development in this cohort, as compared with adult animals, may be relevant to the interpretation of the results, and that decreases in γδ T-cell levels, for example, as animals age, may affect the immune response. As no concurrent diseases were detected and animals were housed and managed in identical conditions throughout the experimental period, we were able to identify a subset of DEGs due to *F. hepatica* infection. Particular attention must be attributed to breed as it is known that differences in animal susceptibility and immunological features vary across breeds. For example, differences in nitric oxide production by macrophages after *in vitro* stimulation of the Brown Swiss cattle breed compared to Holstein-Friesian have been demonstrated (44) and relative resistance of the Indonesian thin tail sheep breed against *F. gigantica* (45) has been documented. Changes in global gene expression involved in some biological pathways, including immune responses, from different cattle breeds in leukocytes (46) have also been demonstrated. In our study, 3,410 DEGs were identified between Holstein-Friesian and crossbred Holstein-Friesian cattle, and the MDS plot also confirmed breed as a significant driver of variation. However, this breed effect could be mitigated in the experimental design as equal numbers of animals of each breed per group were included. As there were no significant differences in the liver fluke burden between the two breeds (*P* = 0.405) no further evaluation of the DEGs between the Holstein-Friesian and crossbred Holstein-Friesian groups in response to infection was conducted. However, further studies should be designed, with more biological replicates per breed, in order to confirm whether breed should be considered as an important factor in investigating response to *F. hepatica* infection. This requirement is further highlighted by differences between the two breeds in terms of their lymphocyte eosinophil counts at specific timepoints.

The clinical consequences of fasciolosis differ markedly between cattle and sheep, with the latter suffering potential acute consequences, and mortality, whereas in cattle disease is usually chronic (2). In comparing the results of this study with those in a previous study in sheep (20), parallel differences between the transcriptomic responses in the two species are striking. It is not possible to conclude definitively that these differences do not arise, at least in part, from variation in experimental protocol (comparative level of challenge, for example). However, in neither case the level of challenge was sufficient to produce clinical signs, so we suggest that this evidence indicates two clear differences in the response to *F. hepatica* between cattle and sheep. The first key difference is the proportion of DEGs detected at acute vs. chronic phases with *F. hepatica* responsible for almost 5% of the total DEGs at the acute phase in cattle whereas in sheep this percentage increases to more than 70% for the same time period (19, 20). In cattle, most of the DEGs were observed at the chronic stage of infection. This indicates that when immature flukes penetrate the gut and migrate through the peritoneum of cattle, relatively few changes in gene expression in PBMC are triggered. However, when flukes are migrating through the liver parenchyma and into the bile ducts, they induce a greater number of gene-expression changes. This accurately reflects the different clinical presentations of fasciolosis between the two species. Whereas, sheep are susceptible to acute disease that can be fatal, fasciolosis in cattle has a much more muted clinical presentation, where animals in most cases appear clinically-normal even though they may experience production losses (47) and bystander immunoregulation (12, 48). The second key difference is the magnitude of the log_2_FC in response to infection. In cattle, log_2_FC in DEGs at the different time-points varied from −1 to 3 whereas in sheep log_2_FC up to 22 in PBMCs (20) and 14 in liver tissue (18) were observed. These results also reflect differences in the disease phenotype between these two host species. A study in water buffalo found DEGs with log_2_FC varying from −5 to 5 from liver tissue at chronic stages of *F. gigantica* infections (49), comparable to the magnitude of log_2_FC detected in the present study.

Apart from the two main differences between cattle and sheep outlined above, a combination of information from the DEGs, canonical pathways, upstream regulators, and regulatory effects detected pinpoint immunological events in bovine fasciolosis.

### Polarized T-Helper and Immune Responses

This study shows that the Th2 response pathway is enriched after only 1 week of *F. hepatica* infection. Due to the low numbers of DEGs found at W1, no clear mechanisms related to T-cell responses can be detected at this acute stage of infection, but more information can be deduced from effects after chronic infection. For instance, upregulation of the *IL6* and *IL4I1* genes and involvement of IL-4 signaling pathways is characteristic of a skewed Th2 response (50) and this response is typically driven by chronic *F. hepatica* infection (48). The fact that the activation status of IL-10 as an upstream regulator changed from an activated trend at W1 toward an inhibitory trend at W14 suggests that during the acute phase the immune response in cattle is a more balanced Treg-Th2 response, with increased polarization toward a Th2 response during chronic stages.

The expression of the intercellular adhesion molecule 1 gene (*ICAM1*), which contributes to recruitment of circulating leukocytes to infection sites and stabilizes cell-target interactions (51), was also upregulated at W14. The same effect was observed in buffaloes but at the acute phase of the infection (49). This could reflect that at chronic stages, there is a need to recruit cells to the site of parasite development to clear the infection. The soluble molecules sICAM-1 have been seen to be increased in complicated cases of humans infected with *F. gigantica* (52).

The class II major histocompatibility complex (MHC-II) transactivator gene (*CIITA*) is downregulated at the chronic stage of the infection. The CIITA protein is known to be a positive regulator of MHC-II gene transcription (53). Consequently, downregulation of *CIITA* could explain the fact that gene expression of the MHC-II DO beta gene (*BOLA_DOB*) is downregulated at the same period (log_2_FC = −0.323). Therefore, antigenic components from *F. hepatica* could interfere with maturation of antigen presentation cells and in the cross-talk to CD4 T-cells in cattle. Suppression of MHC-II has been described as an effect of fasciolosis in murine peritoneal dendritic cells (54, 55) and in water buffalo (49).

We observed upregulation of *TLR2, TLR4*, and downregulation of *TLR10* at the chronic stages of infection. This is consistent with previous findings—for example, increased expression of *TLR2* gene by PBMC from *F. hepatica*-infected cattle for 8 weeks (56), of TLR4 protein by the mucin-derived peptide from *F. hepatica* (57) and downregulation of *TLR10* gene in ovine PBMC (20). Nevertheless, Toll-like receptor signaling had a trend of inhibition in the time period from the acute to chronic fasciolosis, suggesting that *F. hepatica* could somehow overcome and interfere in the downstream effect triggered by increased *TLR2* and *TLR4*. The suppression of *TLR4* and TLR functions by antigenic components such as one of the *F. hepatica* fatty acid binding proteins (58) and glycoconjugates (59) has previously been reported. We also observed the activation of *TLR7* in the time period from the acute to chronic fasciolosis in cattle. Activation of *TLR7* gene has been reported in chronic stages of *F. gigantica* infection in water buffalos (60). The TLR7 receptor protein has been reported to mediate early innate immune responses in malaria (61) and it can contribute to the proliferation and activation of B cells and promotion of Th2 responses.

### Liver Fibrosis

**Figure 7:**
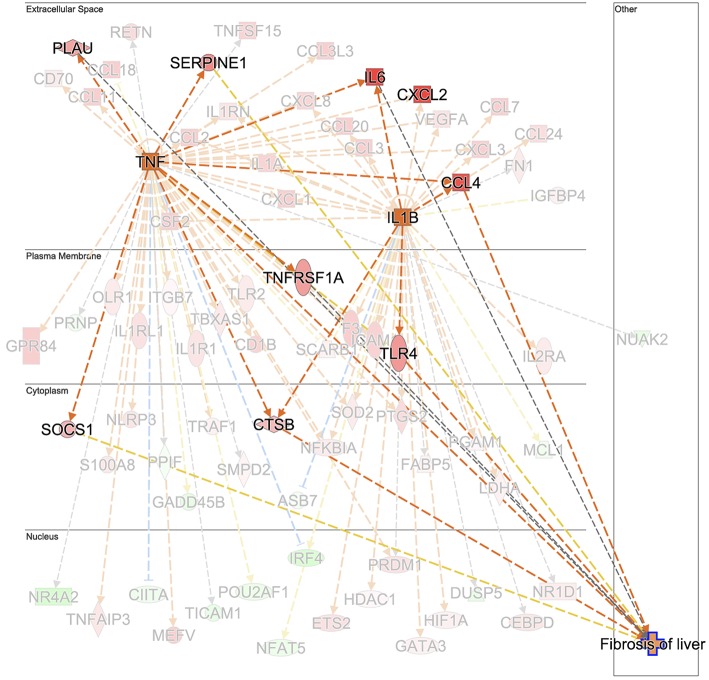
Prediction of *TNF* and *IL1B* as the upstream regulator cascade involved in hepatic fibrosis in animals experimentally infected with *F. hepatica* at 14 weeks post-infection. *TNF* is indicated as activated (orange square). Downstream target genes are highlighted as upregulated (red) or downregulated (green) with color intensity increasing with degree of log_2_ fold-change. Genes are illustrated according to the subcellular location of proteins translated from respective genes. Downstream target genes that do not have an effect in liver fibrosis are shaded in the background. Arrowheads at the end of interactions (dotted lines) indicate activation, while bars indicate inhibitory effects. The colors of lines represent predicted relationships based on gene expression, including orange (activation), blue (inhibition), yellow (findings inconsistent with state of downstream molecule), and gray (effect not predicted). © 2000–2019 QIAGEN. All rights reserved.

Hepatic fibrosis and hepatic cholestasis are clear clinical features seen in chronic fasciolosis. Previous studies have shown that TGFB1 is one of the main molecules orchestrating hepatic fibrosis after *F. hepatica* infection in sheep (18, 20, 62) and in cattle (63). Here, we detected the downregulation of *TGIF1* as early as 1 week post-infection. This gene encodes a repressor of *SMAD2* which leads to a controlled cell proliferation in acute liver injury (64). The plasminogen activator inhibitor-1 (*PAI-1*), which encodes a molecule with high fibrotic activity (65), was detected in ovine fasciolosis (20), and upregulated after 14 weeks of infection. Upregulation of *TGFB1, TGFB2, COL1A1*, and *COL3A1* were found at W14 post-infection but in both infected and uninfected groups, suggesting that there may be alternative mechanisms for hepatic fibrosis in liver fluke-infected cattle. We suggest that the “fibrosis of liver” downstream effect could have been initiated at the chronic stage by the upregulation of *TNF* and that *TGFB1* would contribute to the fibrotic effect at more advanced stages. Further work comparing *TNF* and *TGFB1* changes from W14 onwards of *F. hepatica* infection would clarify the fibrosis events in cattle. TNF leads to the upregulation of the *TNF* gene, and the *IL6* (66–68), urokinase-type plasminogen activator (*PLAU*) (69), *SERPINE1, TNFRSF1A, SOCS1*, and cathepsin B (*CTSB*) genes responsible for the fibrosis downstream effect (Figure 7). In addition, TNF activates *IL1B*, which also contributes to the upregulation of *TLR4* and *IL6*. Upregulation of *IL1B* in water buffalos after 10 weeks of *F. gigantica* infection has previously been observed (49). Upregulation of *TNF* is also consistent with previous findings in sheep PBMC (20) and liver tissue (18) but not with other *in vitro* studies following incubation of *F. hepatica* antigens with human cells (70), murine cells *in vivo* and *in vitro* (58) and the bovine macrophage cell line BOMA *in vitro* (71). Discrepancies in TNF production between studies could be due to the differences in cell type populations when evaluated *in vitro* and *in vivo*, differences in parasite susceptibility between hosts and the antigen composition employed in each case. The activation of hepatic stellate cells by IL6/STAT3 to promote liver fibrosis has been documented in *F. hepatica* infection in humans and mice (72) which is in line with our findings in terms of the activation status of the IL6 and STAT3 signaling pathways at W14.

### Apoptosis

*Fasciola hepatica* infection is known to induce apoptosis in peritoneal leukocytes (73), as well as in liver eosinophils (74), and in PMBC (20, 21) in sheep. In the case of ovine PBMC, the activation of the extrinsic apoptosis pathway by the up-regulation of *TNF* and *TNFR1*/*TNFR2* genes (20) and the potential association of the intrinsic apoptosis pathway with the BH3-only member genes *BIK, BNIP1*, and *BLC2L11* (21) has been postulated. In this study, apoptosis, and death receptor signaling pathways trended toward inhibition at W14. A close analysis reveals that activation of apoptosis of antigen presenting cells is one of the clearest regulatory effects, seen in combination with the migration of mononuclear leukocytes and migration and recruitment of phagocytes, orchestrated by the inhibitory effect of DUSP1 and mir-155 and by the activation of STAT3 and APP regulators. Although activation of STAT3 is known to reduce apoptosis, it could contribute to the upregulation of *IL1B* in combination with APP, mir-155, and DUSP1. Although *DUSP1* was not detected as a DEG in this study it did trend toward upregulation (Log_2_FC = 0.263, FDR = 2.94E-01). Moreover, *DUSP4* and *DUSP16*, were also observed to be upregulated. Other members of the DUSP gene family were upregulated in ovine PMBC at 2 and 8 weeks post-infection, respectively (19). Upregulation of *DUSP13* has been shown in a bovine macrophage cell line stimulated with ES products from two *F. hepatica* isolates (71). *DUSP13* inactivates important cell signaling mediators (JNK, p38, and ERK kinases as well as the transcription factor AP-1), which may lead to reduced inflammation (75).

## Conclusions

In conclusion, this is the first in-depth transcriptomic analysis conducted in cattle for the assessment of the immune response against *F. hepatica* infection. This study pinpoints significant differences in terms of numbers of DEGs and the magnitude of transcriptional responses between cattle and sheep at the acute and chronic stages of infection. These differences are consistent with the different presentations of infection in these two species. It also predicts the potential role of TNF, IL-6, and IL-1B, in the development of early hepatic fibrosis events where TGF-β is not the key player. Apoptosis of antigen presenting cells is orchestrated by mir-155 and the products of the *STAT3, APP*, and *DUSP1* genes at the chronic stages, demonstrating novel mechanisms triggering apoptosis during *F. hepatica* infection in cattle. This analysis is of pivotal importance for the better understanding of the immune response against *F. hepatica* in cattle, and for the development of novel *Fasciola* vaccines, where overcoming parasite-immunoregulatory strategies will be key to success.

## Data Availability

The datasets generated for this study can be found in the European Nucleotide Archive Repository|PRJEB32022.

## Author Contributions

AG-C, JB, and GM conceived and designed the experiments. AG-C, AN-L, and LG-C collected samples from animals and carried out PBMC isolation. AG-C performed lab work and analyzed data. JB supported lab work. CC, GF, and DM assisted in data analyses and bioinformatics. AG-C and GM contributed to the writing of the manuscript. GM, JB, CC, GF, and DM contributed to the editing of the manuscript critically for important intellectual content. All authors read and approved the final manuscript.

### Conflict of Interest Statement

The authors declare that the research was conducted in the absence of any commercial or financial relationships that could be construed as a potential conflict of interest.

## Supplementary Material

Supplementary Figure 1 Evaluation of liver capsule, parenchyma and bile ducts in livers of uninfected (top) and infected (bottom) animals. Fibrotic areas and thickness of bile ducts are indicated by arrows.

Supplementary Figure 2 Expression density of gene counts for the individual samples in each time point after filtering. This plot shows that each sample follows the same trend thus there is no evidence of any poor quality or outlier libraries.

Supplementary Figure 3 Venn diagrams showing the numbers of DEGs in identified in PBMC and their direction of expression in uninfected and *F. hepatica* infected animals. Comparison of DEGs from W1 vs. W0 **(A)**, W14 vs. W0 **(B)** and W14 vs. W1 **(C)**. Downregulated genes are shown in gray and pink, upregulated genes in green and blue.

Supplementary Table 1 Reads from pooled RNA-seq libraries uniquely mapping to the bovine genome.

Supplementary Table 2 Confirmation of identification of samples corresponding to each individual animal.

Supplementary Table 3 DEGS identified between Holstein-Friesian and Holstein-Friesian crossbreds.

Supplementary Table 4 DEGs identified between the W0, W1, and W14 timepoints.

Supplementary Table 5 DEGs identified exclusively in infected animals between each timepoint comparison, and hence driven by *F. hepatica* infection.

Supplementary Table 6 RT-qPCR validation of six genes from the DEG dataset.

Supplementary Table 7 Enriched canonical pathways identified by IPA analysis.

Supplementary Table 8 Activation status of upstream regulators identified in comparisons of W1 vs W0 **(A)**, W 14 vs. W0 **(B)** and W14 vs. W1 **(C)**.
