# Supplementary figures and images for: Fasciola hepatica Infection in Cattle: Analyzing Responses of Peripheral Blood Mononuclear Cells (PBMC) Using a Transcriptomics Approach
Source: Front Immunol. 2019 Aug 29;10:2081. doi: 10.3389/fimmu.2019.02081 (PMC6727689; doi:10.3389/fimmu.2019.02081)

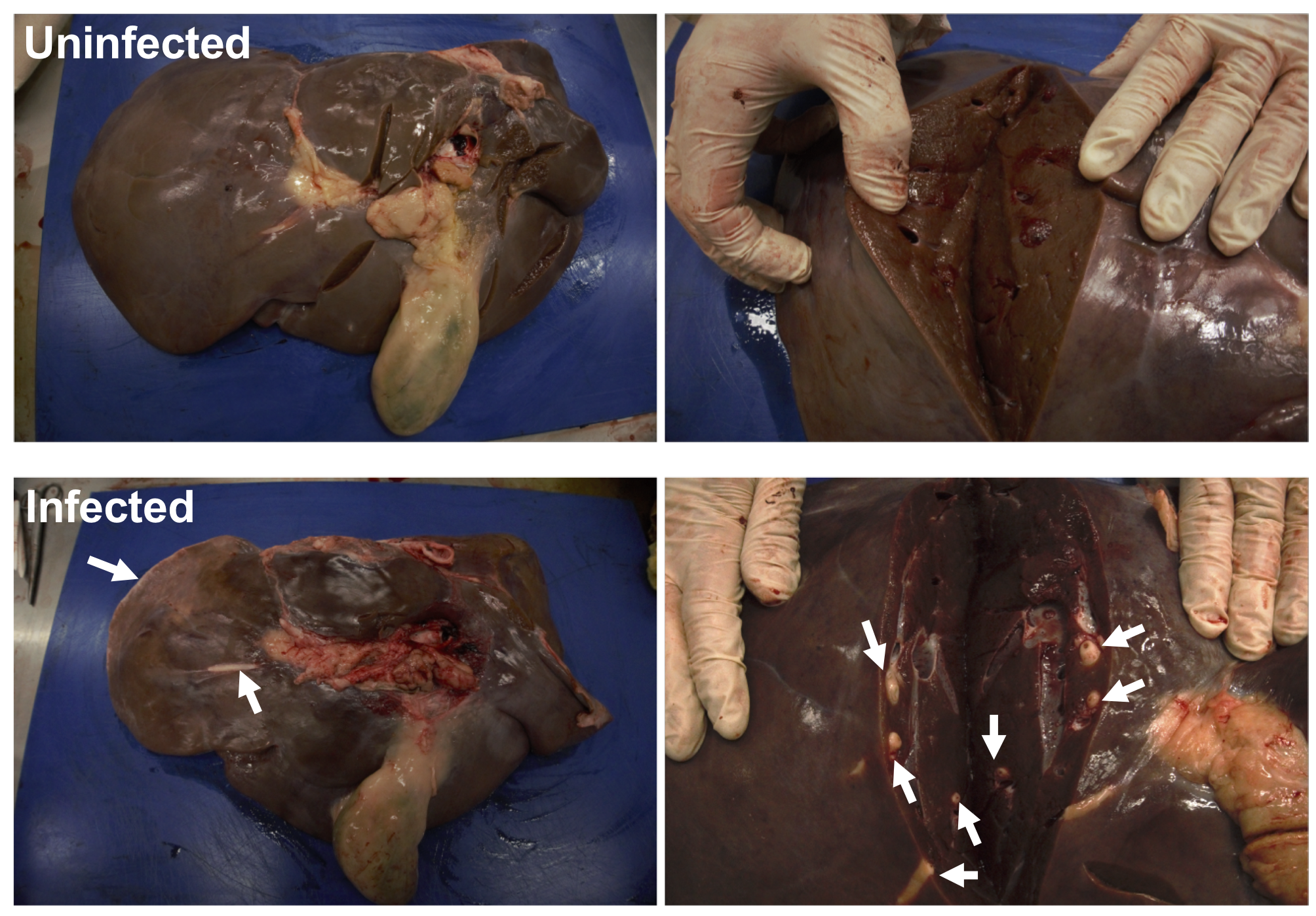

Supplement: Supplementary Figure 1 — Evaluation of liver capsule, parenchyma and bile ducts in livers of uninfected (top) and infected (bottom) animals. Fibrotic areas and thickness of bile ducts are indicated by arrows. [file Image_1.tiff]

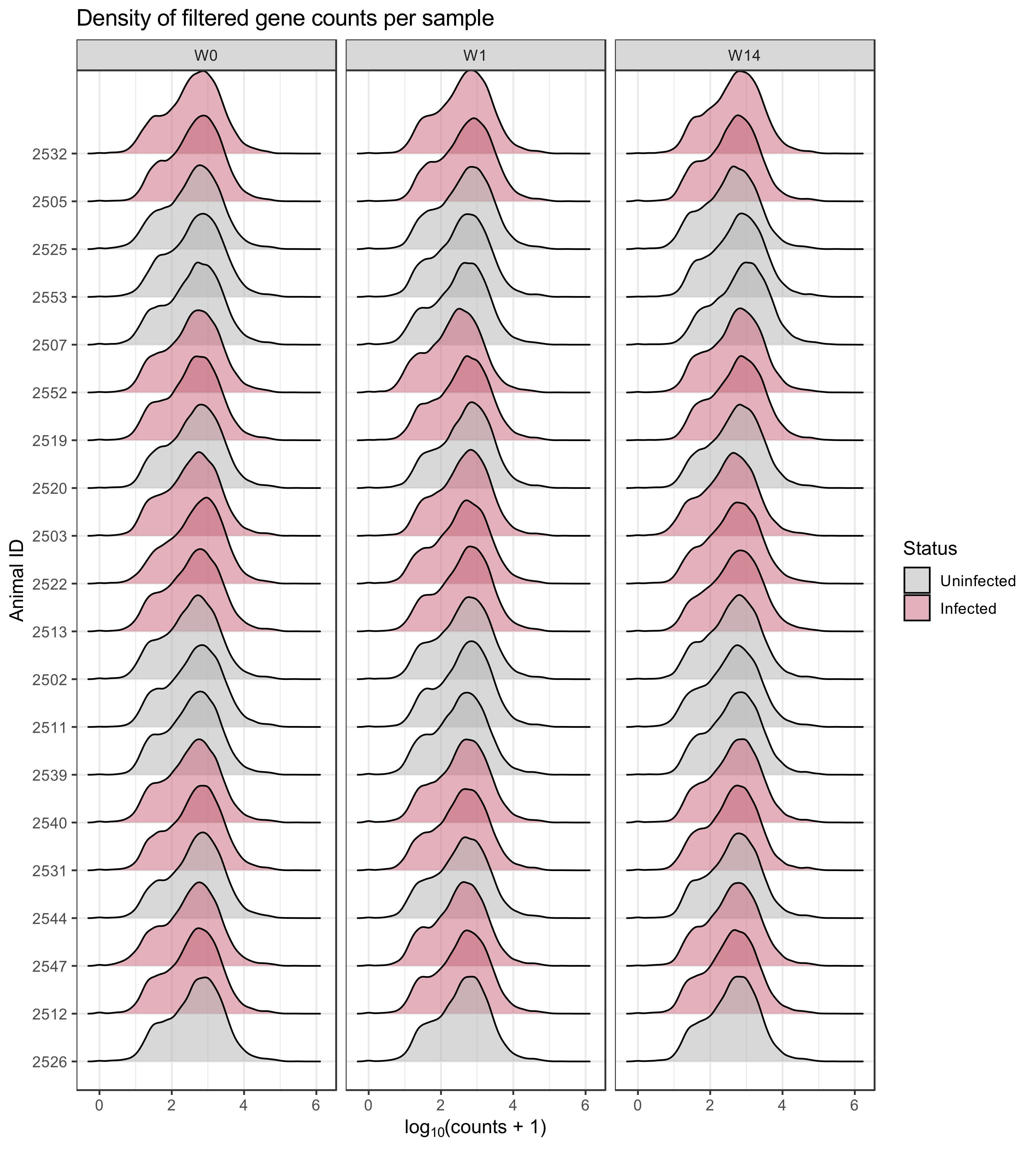

Supplement: Supplementary Figure 2 — Expression density of gene counts for the individual samples in each time point after filtering. This plot shows that each sample follows the same trend thus there is no evidence of any poor quality or outlier libraries. [file Image_2.tiff]

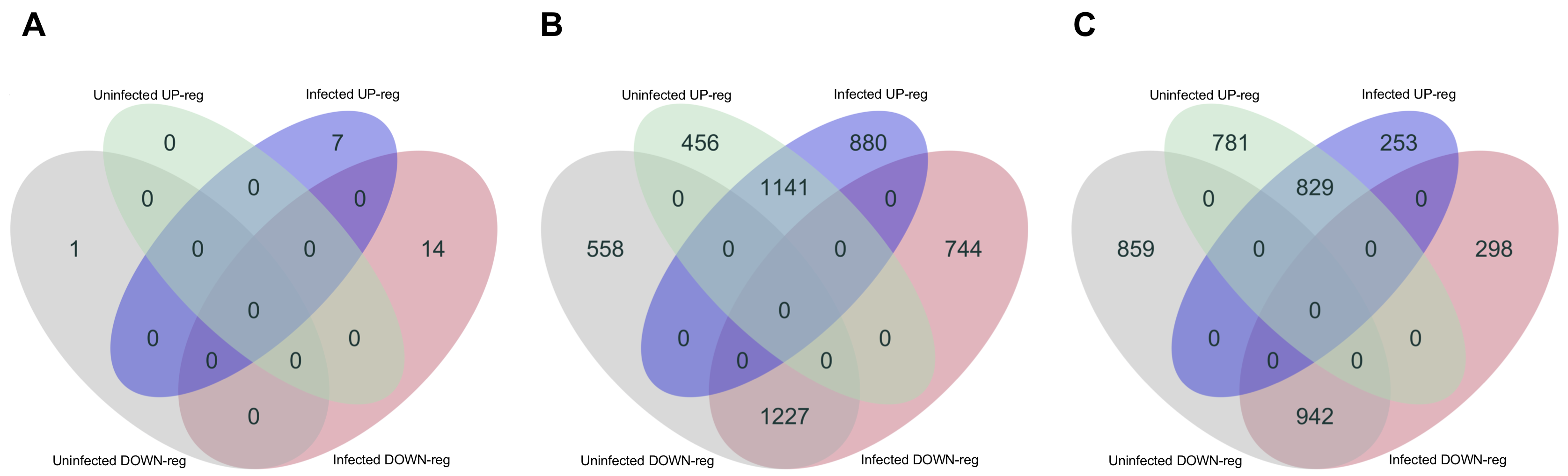

Supplement: Supplementary Figure 3 — Venn diagrams showing the numbers of DEGs in identified in PBMC and their direction of expression in uninfected and F. hepatica infected animals. Comparison of DEGs from W1 vs. W0 (A), W14 vs. W0 (B) and W14 vs. W1 (C). Downregulated genes are shown in gray and pink, upregulated genes in green and blue. [file Image_3.tif]
